# Selective detection of methanol vapour from a multicomponent gas mixture using a CNPs/ZnO@ZIF-8 based room temperature solid-state sensor†

**DOI:** 10.1039/d2ra04665b

**Published:** 2022-09-23

**Authors:** Lesego Malepe, Derek Tantoh Ndinteh, Patrick Ndungu, Messai Adenew Mamo

**Affiliations:** Department of Chemical Science, University of Johannesburg PO Box 17011, Doornfontein 2028 Johannesburg South Africa messaim@uj.ac.za; Department of Chemistry, University of Pretoria Private Bag X20, Hatfield 0028 Pretoria South Africa patrick.ndungu@up.ac.za

## Abstract

Methanol vapour is harmful to human health if it is inhaled, swallowed, or absorbed through the skin. Solid-state gas sensors are a promising system for the detection of volatile organic compounds, unfortunately, they can have poor gas selectivity, low sensitivity, an inferior limit of detection (LOD), sensitivity towards humidity, and a need to operate at higher temperatures. A novel solid-state gas sensor was assembled using carbon nanoparticles (CNPs), prepared from a simple pyrolysis reaction, and zinc oxide@zeolitic imidazolate framework-8 nanorods (ZnO@ZIF-8 nanorods), synthesised using a hydrothermal method. The nanomaterials were characterized using scanning electron microscopy, transmission electron microscopy, powder X-ray diffraction, X-ray photoelectron spectroscopy Raman spectroscopy, and Fourier transform infrared spectroscopy. The ZnO@ZIF-8 nanorods were inactive as a sensor, the CNPs showed some sensor activity, and the CNPs/ZnO@ZIF-8 nanorod composite performed as a viable solid-state sensor. The mass ratio of ZnO@ZIF-8 nanorods within the CNPs/ZnO@ZIF-8 nanorod composite was varied to investigate selectivity and sensitivity for the detection of ethanol, 2-propanol, acetone, ethyl acetate, chloroform, and methanol vapours. The assembled sensor composed of the CNPs/ZnO@ZIF-8 nanorod composite with a mass ratio of 1.5 : 6 showed improved gas sensing properties in the detection of methanol vapour with a LOD of 60 ppb. The sensor is insensitive to humidity and the methanol vapour sensitivity was found to be 0.51 Ω ppm^−1^ when detected at room temperature.

## Abstract 2

Methanol vapour is harmful to human health if it is inhaled, swallowed, or absorbed through the skin.

## Graphical abstract

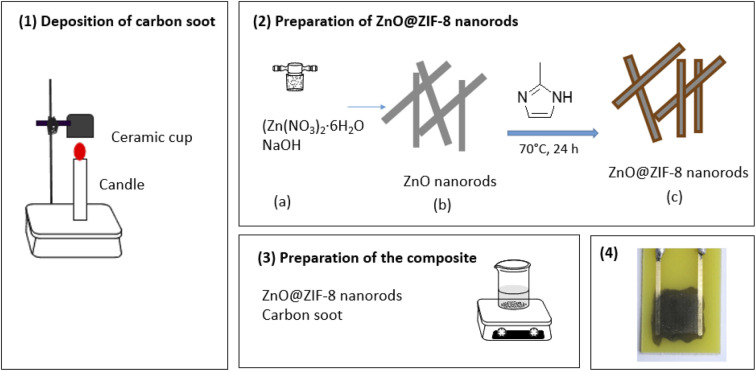

## 1. Introduction

Methanol (CH_3_OH) is an organic compound that can easily evaporate at lower temperatures due to its high vapour pressure and low boiling point.^1^ It is a type of volatile organic compound (VOC) that is used in several applications including food technology,^2^ synthesis of various commercially important organic compounds,^3^ paint industries, automobile manufacturing, and pharmaceuticals.^4–6^ However, the inhalation of methanol can be dangerous to human health as it causes eye irritation, headaches, and can have deleterious effects on the nervous system.^6–8^ As a replacement for fossil fuels, CH_3_OH is considered to be a promising candidate to be used in automobiles, especially those fitted with fuel cells. Since it is flammable, there is a need to monitor the leakage of methanol from on board tanks using a relatively simple system, such as, gas sensors.^9,10^ There is a high rate of car accidents resulting from drunk drivers, especially in South Africa, but with the advance in technology, gas sensors can be used to identify drunk drivers through the detection of either methanol or ethanol vapours from their exhaled breath.^11,12^ In the past, methanol vapour and other VOCs were detected using highly accurate, but relatively complex, analytical instruments including gas chromatography,^13^ spectrophotometers,^14^ and optoacoustic spectroscopy.^15^ But such instruments pose some disadvantages to being effectively used on a much wider scale, due to their high costs, lack of portability, need for trained personnel, long analysis time, and expensive maintenance requirements. Recently, chemi-resistive gas sensors have attracted many researchers and engineers to develop and use them in the detection of VOCs due to their portability, low-cost, high sensitivity, and fast analysis time. Semiconductor metal oxides (SMOs) such as titanium dioxide (TiO_2_),^16^ zinc oxide (ZnO),^17^ tin dioxide (SnO_2_),^18^ and tungsten trioxide (WO_3_)^19^ are some of the earliest studied and applied materials in chemi-resistive gas sensors. This can be attributed to their fast response–recovery times and high sensitivity. However, such metal oxides have some drawbacks including the lack of selectivity towards the gas of interest, poor response under high humidity conditions, and a high working temperature of 240–400 °C.^16–19^ The high working temperature is a major drawback because it leads to increased energy consumption and this results in a poor lifespan of the sensor since the material used to fabricate the sensor disintegrates after being repeatedly exposed to such conditions. To overcome some of these drawbacks, SMOs can be coupled with polymers such as polyaniline,^20^ polypyrrole, and polythiophene.^21^ Alternatively, carbon nanomaterials including carbon nanotubes,^22^ graphene oxide,^23^ and carbon nanoparticles^24^ are another promising option to make hybrids that can be used as sensors that work at a lower temperature. SMO nanocomposites can have a good response–recovery times and high sensitivity; however, selectivity towards the gas of interest and humidity effects are still major challenges with such hybrid materials.

Zeolitic imidazolate frameworks (ZIFs) are a subclass of a metal–organic framework materials with a tetrahedral arrangement between the metal ion and imidazolate linker, possessing a geometry similar to that of zeolites.^25–28^ ZIFs materials have been used in gas sensors, due to their porosity and intrinsic tunability which has improved selectivity. In addition, their hydrophobic nature allows the sensor to work at relatively high levels of humidity.^25–30^ ZIF-8 is composed of zinc cation and 2-methyimidazolate precursors and is reported to be chemically and thermally stable.^31,32^ ZIF-8 has been combined with an SMO to make gas sensors; specifically, ZnO@ZIF-8 nanorods were synthesised and shown to have good selectivity, sensitivity, and fast response recovery times. However, such gas sensors still operate at elevated temperatures of about 140 to 350 °C.^33,34^

In this study, ZnO@ZIF-8 nanorods and carbon nanoparticles (CNPs) were used as a composite for the detection of methanol at room temperature. Besides being an inexpensive and versatile nano-carbon, our previous work with CNPs derived from candle soot in gas sensing has shown that such sensors have an excellent response–recovery times and the ability to work at room temperature.^35–38^ The proposed CNPs/ZnO@ZIF-8 nanorods composite can work at room temperatures, possess high sensitivity, selectivity, longer life-span, good reproducibility, fast response–recovery time, and the material can work and reproduce the same results at a wide range of relative humidity.^39^

## 2. Experimental

### 2.1. Chemicals and reagents

All chemicals were used without further purification: zinc nitrate hexahydrate (Zn(NO_3_)_2_·6H_2_O, 98%), sodium hydroxide (NaOH, 96%), 2-methylimidazole (99%) and *N*,*N*-dimethylformamide (DMF, 99.5%), methanol (MeOH, 99.8%), ethanol (EtOH, 98%), 2-propanol (98.5%), chloroform (99%), ethyl acetate (99.8), and acetone (99.5%) were all purchased from Sigma Aldrich. Lighthouse candles were purchased at Shoprite, Johannesburg, South Africa.

### 2.2. Synthetic methods

#### 2.2.1. Carbon nanoparticles

**Fig. 1:**
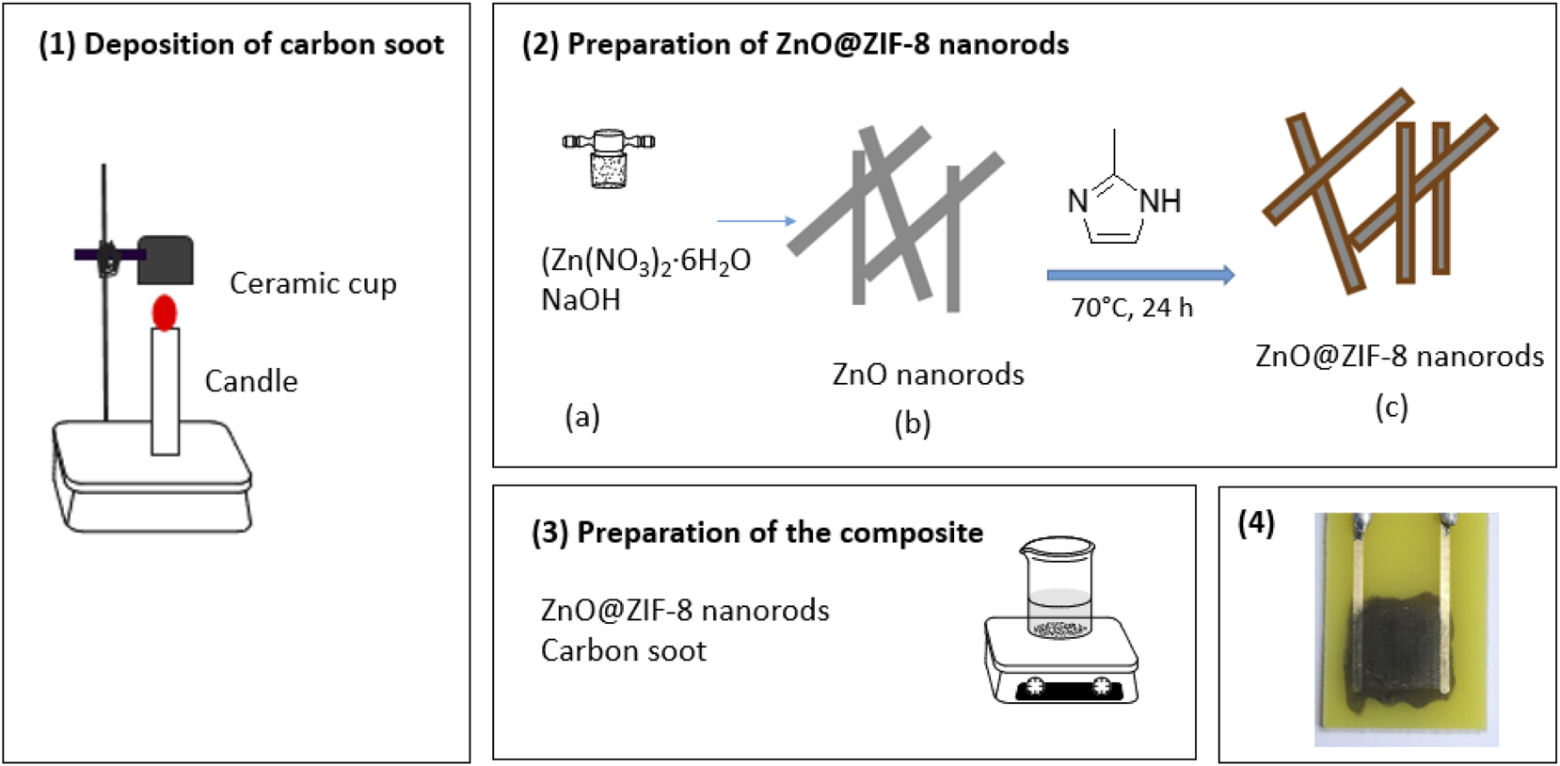
Schematic illustration of (1) preparation of CNPs, (2) synthesis of ZnO@ZIF-8 nanorods and CNPs/ZnO@ZIF-8 nanorods composite preparation.

Carbon soot was prepared through the pyrolysis method following the method reported by G. E. Olifant *et al*.^24^ The candle was placed underneath the ceramic cup (approximately 2 cm from the tip of the flame) for the collection of carbon soot as shown in (Fig. 1). The ceramic cup was cooled at room temperature and a spatula was used to scrape the accumulated carbon soot from the inside of the ceramic cup walls. The soot was used without purification.

#### 2.2.2. Synthesis of ZnO@ZIF-8 nanorods

ZnO@ZIF-8 nanorods were synthesised *via* the solvothermal method reported by H. Tian *et al.*,^34^ whereby ZnO nanorods acted as a substrate for the coating of 2-methylimidazolate. Firstly, a hydrothermal method was followed for the preparation of ZnO nanorods. In a typical procedure, 2.26 g of Zn(NO_3_)_2_·6H_2_O and 2.43 g of NaOH were both added to a beaker with 40 mL of deionized water. The white solution was stirred using a magnetic stirrer and stirrer bar for 30 min, and then the solution was transferred into a Teflon lined-stainless steel autoclave. The autoclave was placed in an oven set at 140 °C, and the reaction was left for 12 hours. The resulting ZnO nanorods were then obtained through centrifugation and washing several times with ethanol and deionized water. The final washed white precipitates were dried, in an oven, at 60 °C for 12 hours.

To prepare the ZnO@ZIF-8 samples, 0.0407 g of ZnO nanorods and 0.324 g of 2-methylimidazolate were mixed in a beaker containing 40 mL of DMF : H_2_O (3 : 1 by volume). The solution was sonicated for 5 min and then transferred into a Teflon-lined stainless steel autoclave and heated at 70 °C for 24 hours. Subsequently, the ZnO@ZIF-8 nanorods were collected through centrifugation, washed several times with ethanol and DMF and dried at 60 °C for 8 h.

#### 2.2.3. Synthesis of CNPs/ZnO@ZIF-8 nanorods composite

CNPs/ZnO@ZIF-8 nanorods composite was prepared through physical mixing. A mass of 0.1 g of CNPs and 0.6 of ZnO@ZIF-8 nanorods were mixed in 15 mL of DMF. The black solution was stirred at room temperature for 12 h and the composite was obtained by drying at 70 °C for 24 h (see Fig. 1).

### 2.3. Sensor fabrication

ZnO@ZIF-8 nanorods, CNPs, and CNPs/ZnO@ZIF-8 nanorods composites were used to fabricate six sensors for the detection of VOCs. 15 mg of ZnO@ZIF-8 nanorods and CNPs were dispersed separately into 7 mL DMF to prepare sensor A and sensor B respectively. Different mass ratios of CNPs and ZnO@ZIF-8 nanorods as composites were used to prepare more sensors, wherein 15 mg CNPs were kept constant throughout and ZIF-8 nanorods weight within the composite was varied to 15 mg, 25 mg, 35 mg, and 60 mg. Sensor C was fabricated from an equal mass ratio (15 : 15 mg) of CNPs : ZnO@ZIF-8 nanorods solution, 15 : 25 mass ratio for sensor D, 15 : 35 mass ratio for sensor E, and 15 : 60 mass ratio for sensor F (see Table 1). All mass ratios were dissolved into 7 mL DMF and stirred for 24 h at room temperature, subsequently, a 7 μL solution of each was drop-coated onto an interdigitated gold electrode and allowed to dry. Prepared sensors were placed in the vacuum desiccator to further dry any remaining DMF left.

**Table 1:** The summarised prepared sensors with their respective material mass ratios

Sensor name	Sensing material	Mass ratio (mg), CNPs : ZnO@ZIF-8 NRs
Sensor A	ZnO@ZIF-8 NRs	0 : 15
Sensor B	CNPs	15 : 0
Sensor C	CNPs : ZnO@ZIF-8 NRs	15 : 15
Sensor D	CNPs : ZnO@ZIF-8 NRs	15 : 25
Sensor E	CNPs : ZnO@ZIF-8 NRs	15 : 35
Sensor F	CNPs : ZnO@ZIF-8 NRs	15 : 60

### 2.4. Gas sensing setup

**Fig. 2:**
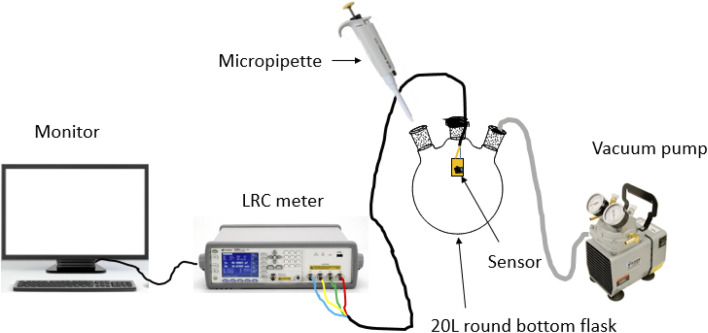
Gas sensing setup.

All sensors were tested using the same sensing setup under the resistance parameter. A sensor connected to the E4980A keysight LCR meter was placed inside a 3-necked 20 L round bottom flask (see Fig. 2). The round bottom flask (sensing chamber) has an opening for a pipe connected to a vacuum pump to remove gas from inside the chamber and it also has a pipe to introduce some external fresh air. Five trials of 1, 2, 3, 4 and 5 μL of VOC of interest were injected into the round bottom flask with a contact time of 10 min, subsequently, the exposed VOCs were removed using a vacuum pump for 2 min at atmospheric pressure and a rest period of 3 min was done before the next measurement. Due to the high vapour pressure and low boiling point of the VOCs, the gaseous analyte interacted with the sensor of interest, and the measurements were observed on the monitor. The concentration of the analytes was calculated using the formula:*C* = (22.4*pTV*_s_)/(273*M*_r_*V*) × 1000,where *C* is the vapour concentration (ppm), *p* is the density of the liquid analyte (g cm^−1^), *T* is the temperature (K), *V*_s_ is the volume injected into the 20 L volumetric flask (μL), *M*_r_ is the molar mass of the liquid analyte and *V* is the volume of the volumetric flask (L).^35,37^

The resistance measurements were recorded using the optimal 0.5 V AC and at a 25 kHz signal frequency. The frequency selection was due to the noise/signal ratio was not significant and the devices responded with good sensitivity.

### 2.5. Sensor's response and recovery test

The sensor's response and recovery time of the sensors were defined as when the response time is the time that required the sensor to reach 90% of the maximum response and while recovery time is that required to recover 90% of the response.

### 2.6. Selectivity test

Firstly, 1 mL of each of the six analytes (methanol, ethanol, 2-propanol, acetone, ethyl acetate, and chloroform) were combined to make 6 mL and mixed well under stirring. From the mixture, a 6 μL aliquot was injected into a 3-necked 20 L round bottom flask to investigate the selectivity of methanol.

### 2.7. Characterization techniques

Samples were analysed on a JEOL-TEM 2010 (Japan) high-resolution transmission electron microscopy at an acceleration voltage of 200 kV, using Gatan software, and Holey carbon-coated copper grids were used to mount the samples. Scanning electron microscopy observations were performed at 30 kV with a FEI Nova Nanolab 600 FEG-SEM. Structural analysis was revealed using powder X-ray diffraction (PXRD), Bruker D2 Phaser using LynxEye detector with radiation of a CuKα at a wavelength of 0.154 nm and Bruker Senterra laser Raman spectrometer fitted with frequency-doubled Nd-YAG laser with the wavelength of 532 nm. X-ray photoelectron spectroscopy (XPS, XSAM800, Kratos, Manchester, UK) was used to determine the oxidation state and the elemental analysis.

## 3. Results and discussions

### 3.1. Materials characterizations

**Fig. 3:**
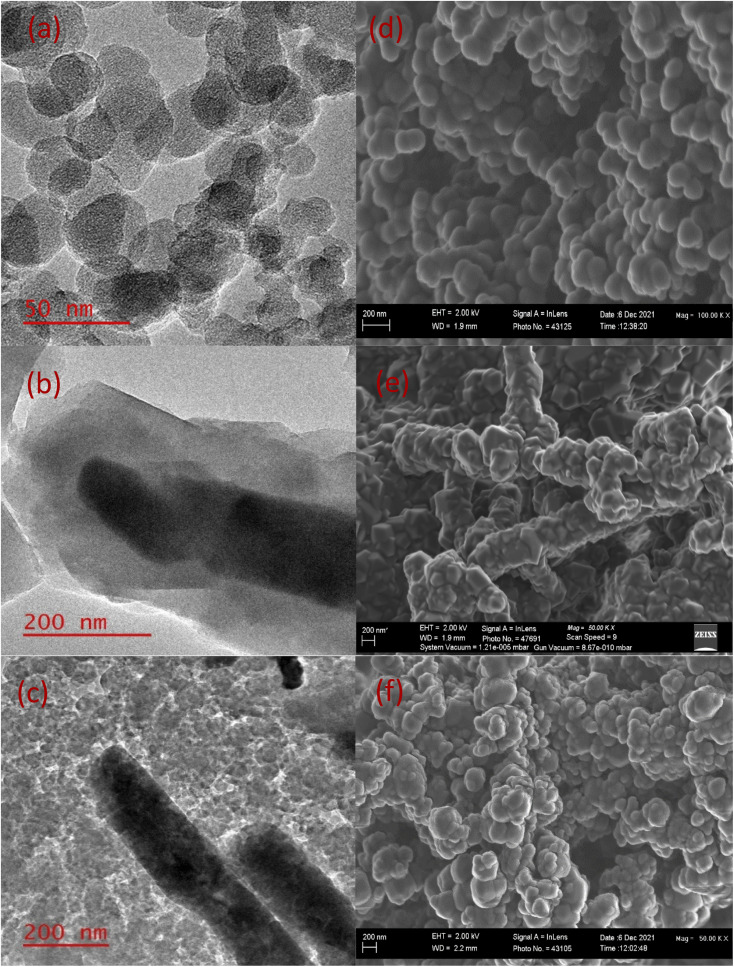
TEM image of (a) CNPs, (b) ZnO@ZIF-8 nanorods and (c) CNPs/ZnO@ZIF-nanorods. SEM image of (d) CNPs, (e) ZnO@ZIF-8 nanorods, and (f) CNPs/ZnO@ZIF-8 nanorods.

Fig. 3 presents the morphological investigations of CNPs, ZnO@ZIF-8 nanorods, and CNPs/ZnO@ZIF-8 nanorods done using electron microscopy. Fig. 3a shows the TEM image of carbon nanoparticles soot, which appears to be spherical with a diameter between 30 and 50 nm. The CNPs soot appears to be stacked on top of each other forming chain-linked spheres obtained from flame pyrolysis, similar findings were reported by L. Malepe *et al.*^36^ The ZnO@ZIF-8 nanorods are prepared using the ZnO nanorods and Zn^2+^ source. The ZnO nanorods are acting as a template and are coated as a shell with a 2-methylimidazole linker to form ZnO@ZIF-8 nanorods. ZIF-8 structure is formed from the coordination of central Zn^2+^ and 1,3 nitrogen positions on the 2-methylimidazole. Fig. 3b presents the image of ZnO@ZIF-8 nanorods with the core–shell heterostructures. The core ZnO nanorods have a diameter of about 140 nm coated with the ZIF-8 shell of about 100 nm and similar findings were reported by H. Tian *et al.*^34^ The TEM image of CNPs/ZnO@ZIF-8 nanorods revealed that the CNPs soot covered the surface of ZnO@ZIF-8 nanorods (see Fig. 3c), which played a crucial role in improving the electrical conductivity that facilitated the gas sensing.

**Fig. 4:**
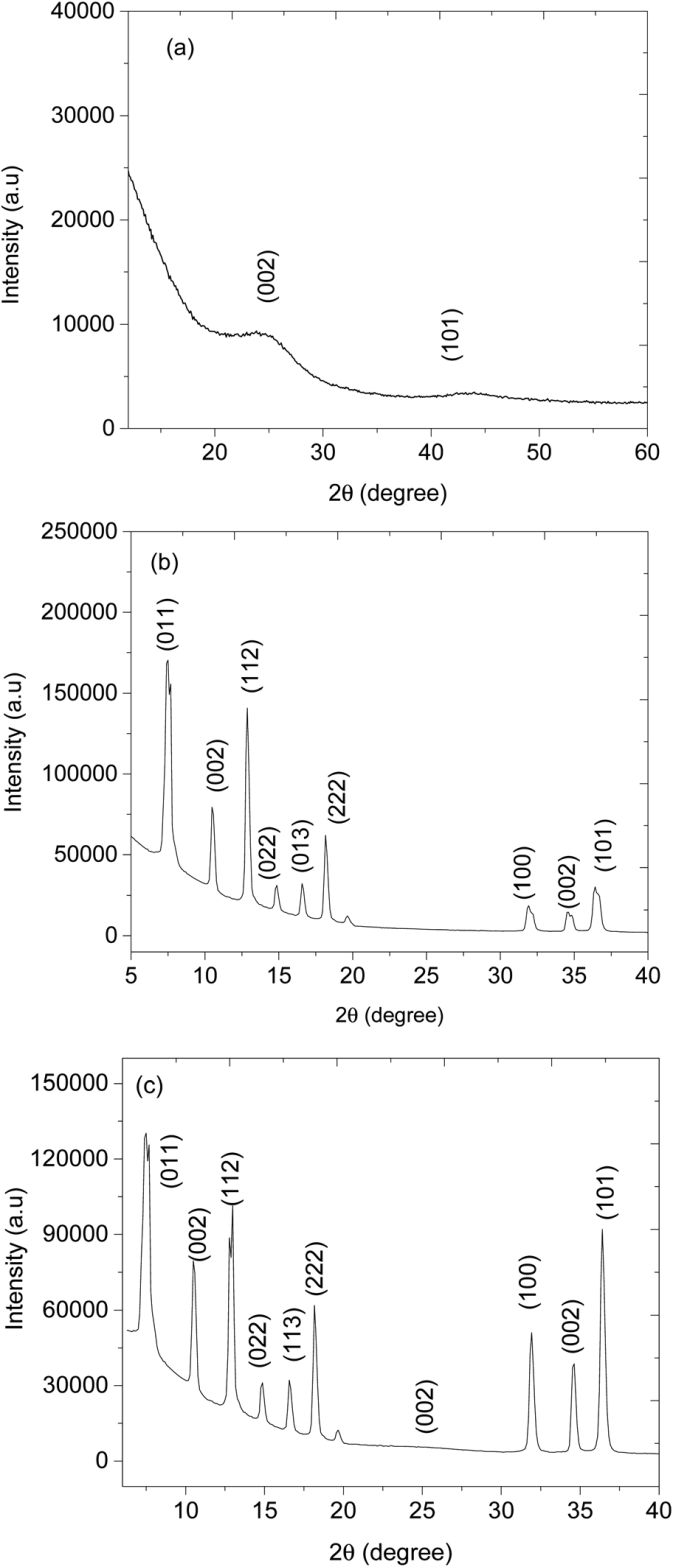
XRD patterns of (a) CNPs, (b) ZnO@ZIF-8 nanorods, and (c) CNPs@ZnO@ZIF-8 nanorods.

The surface morphology of CNPs, ZnO@ZIF-8 nanorods, and CNPs/ZnO@ZIF-8 nanorods composite was investigated using scanning electron microscopy (SEM). Fig. 3d shows an SEM image of the CNPs spheres and reveals that the materials are agglomerated and interconnected forming irregular lumps of microstructures. The ZnO@ZIF-8 nanorod samples occur as tiny rods on the microscale, and in some areas, the nanorods tend to agglomerate forming sphere-flower-like structures as presented in (Fig. 3e). The successful synthesis of CNPs/ZnO@ZIF-8 nanorods was confirmed by the presence of agglomerated CNPs and ZnO@ZIF-8 nanorods on the SEM image shown in (Fig. 3f). The results of the powder X-ray diffraction (XRD) analysis on the CNPs, ZnO@ZIF-8 nanorods and CNPs/ZnO@ZIF-8 nanorods are presented in (Fig. 4). The XRD pattern of CNPs showed two peaks occurring at 2*θ* = 25.2° and 44° assigned to the crystal planes 002 and 001 respectively (ICDD: 04-018-7559) for graphitic carbon. The broad, and relatively low-intensity peaks are typical for CNPs.

**Fig. 5:**
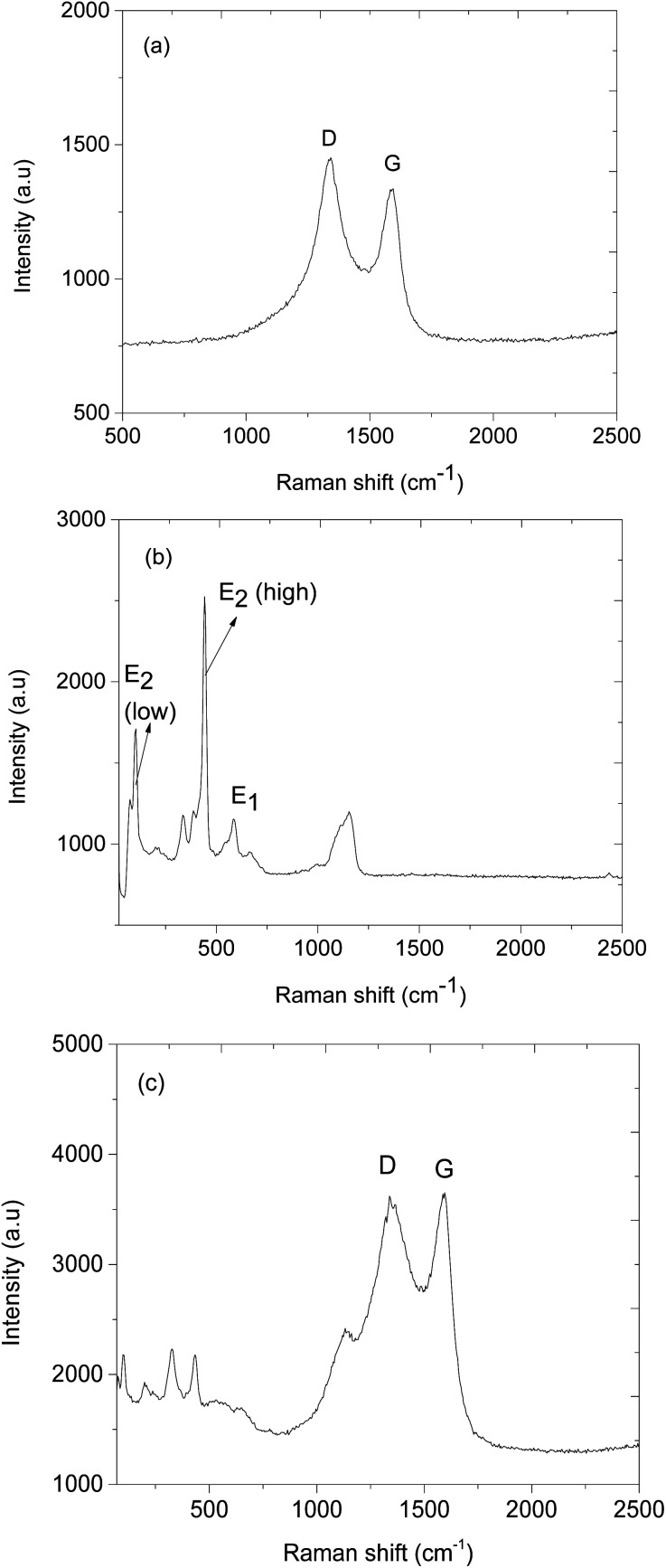
The Raman spectra of (a) CNPs, (b) ZnO@ZIF-8 nanorods, and (c) CNPs/ZnO@ZIF-8 nanorods.

Fig. 4b presents the XRD pattern of ZnO@ZIF-8 which indicates that it has two materials of different crystal structures. The XRD patterns positioned at 2*θ* = 31.9°, 35° and 37.9° are indexed to crystal planes 100, 002 and 101, the reflections of hexagonal structure of ZnO (JCPDS No. 36-1451) and while ZIF-8 XRD patterns match with literature, wherein 2*θ* = 7.6°, 10.4°, 13°, 15.2°, 16.9° and 18.2° are indexed to crystal planes 011, 002, 112, 022, 013 and 222 respectively.^40–44^Fig. 4c, shows the XRD patterns of CNPs/ZnO@ZIF-8 composite, which shows the presence of CNPs by exhibiting the broad peak occurring at 2*θ* = 25.2° (002), however, the peak has a low intensity due to the fact that the high crystallinity of ZnO@ZIF-8 nanorods within the composite suppress the amorphous and graphitic peaks. Fig. 5a, presents the Raman spectrum of CNPs exhibiting two broad peaks at 1344 and 1591 cm^−1^ assigned to D, and G bands respectively. The D band represents the disordered lattice nature of the CNPs while the G band is assigned for the graphitic vibrations (sp^2^) of the CNPs.^45,46^Fig. 5b, presents the Raman spectrum of ZnO@ZIF-8 nanorods and exhibits a vibrational mode positioned at 220 cm^−1^ which is assigned to Z–N stretchings and while others occurring at 679, 1014, 1249, and 1485 cm^−1^ are attributed C5–N stretchings and 2-methyl bending of the 2-methyl imidazolate ring.^47–49^ Several other peaks were observed at 110, 351, 439, 588 and 1149 cm^−1^, and are attributed to vibrational transitions from the ZnO nanorods. ZnO nanorods are Raman active on polar (A1 and E1) and slit into longitudinal optical (LO) and transverse optical (TO) phonons. The hexagonal structure of ZnO is confirmed by a peak occurring at 101 and 439 cm^−1^ are assigned for E_2_ (low) and E_2_ (high) mode and the peak 588 cm^−1^ corresponds to E_1_ (LO) mode that is assigned for the presence of oxygens.^50,51^ The successful synthesis of CNPs/ZnO@ZIF-8 nanorods is confirmed by the appearance of the D and G bands from the CNPs and ZnO@ZIF-8 nanorods Raman vibrations modes (see Fig. 5c).

**Fig. 6:**
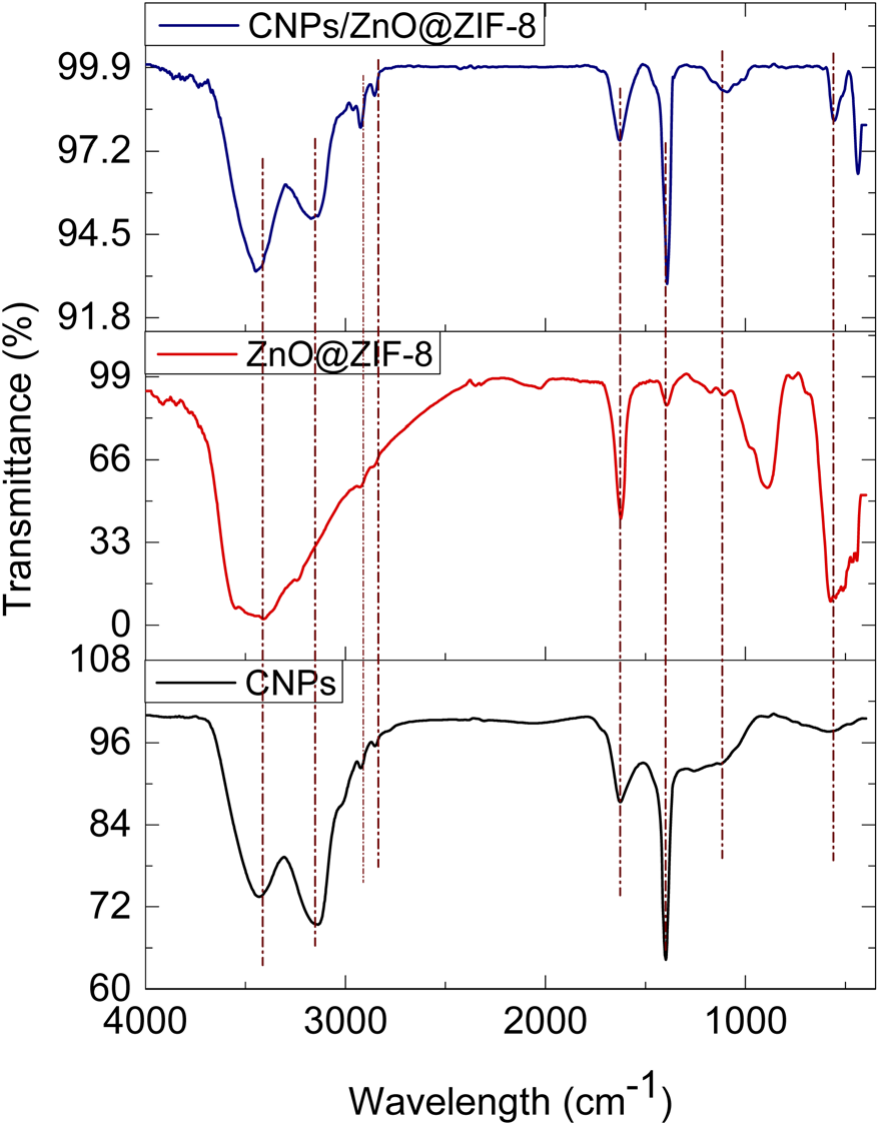
FTIR of CNPs, ZnO@ZIF-8 nanorods, and CNPs/ZnO@ZIF-8 nanorods composite.

The chemical bonding nature of the materials was studied using FTIR presented in (Fig. 6). The FTIR spectrum of CNPs showed a broad peak positioned at 3555 cm^−1^ representing O–H stretching, 3233 cm^−1^ and 3129 cm^−1^ are for the C–H and the peak occurring at 1374 cm^−1^ is for C–O–C bonding character. In addition, the 429 cm^−1^ is due to Zn–N stretching confirming the bonding of the 2-methyl imidazolate linker and Zn, the peaks positioned at 1616 cm^−1^ and 3475 cm^−1^ indicate the presence of surface-adsorbed of H_2_O and O–H on Zn–OH stretching occurring in all synthesized materials. The peak at 550 cm^−1^ confirms the Zn–O–Zn–O bonding mode. The observed peak at 1390 cm^−1^ coming out on CNPs and ZnO@ZIF-8 confirms the C–OH stretching, wherein ZnO@ZIF-8 might have resulted from surface-adsorbed moisture while in CNPs might be resulted during formation.^52,53^ The presence of 429 cm^−1^ and 550 cm^−1^ peaks at CNPs/ZnO@ZIF-8 nanorods spectrum confirms the presence of Zn–N stretching^54,55^ and Zn–O–Zn–O stretching mode respectively attributed to ZnO@ZIF-8 nanorods, and while the peaks at 3233 cm^−1^ and 3129 cm^−1^ confirms the presence the of CNPs within the composite, thus the CNPs/ZnO@ZIF-8 nanorods composite was successfully formed.

**Fig. 7:**
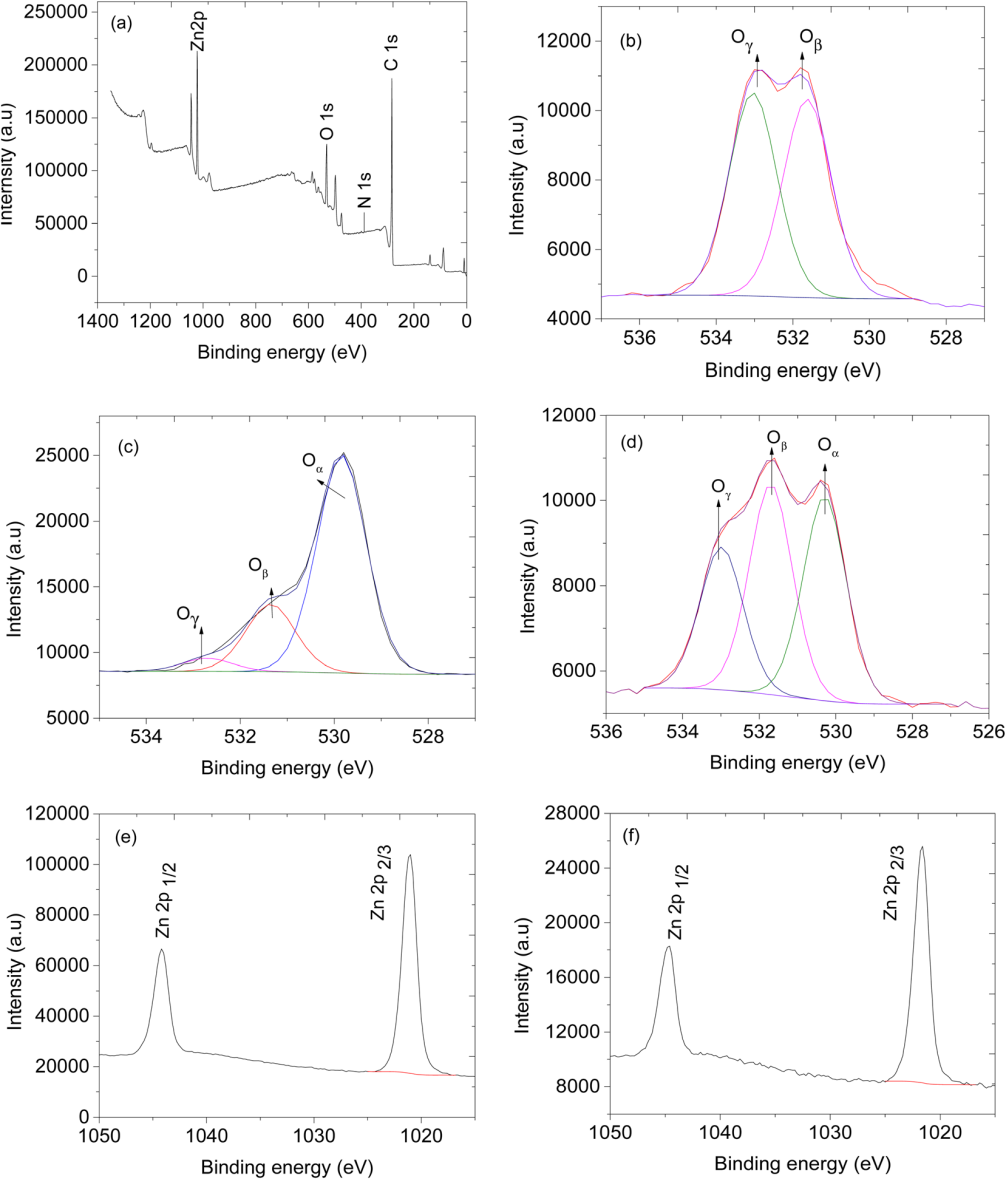
XPS spectra, (a) ZnO@ZIF-8/CNPs survey, (b) O 1s of CNPs, (c) O 1s of ZnO@ZIF-8, (d) O 1s of ZnO@ZIF-8/CNPs, (e) Zn 2p of ZnO@ZIF-8 and (f) Zn 2p of ZnO@ZIF-8/CNPs.

The oxygen species on the surface of the sensing material plays crucial role in gas sensing application.^56^ Therefore, X-ray photoelectron spectroscopy was carried out to identify the type of oxygen species present on the sensing materials, the oxidation state of zinc, and the chemical composition ZnO@ZIF-8/CNPs composite. Fig. 7 shows the XPS survey spectrum presenting the existence of Zn 2p, O 1s, N 1s and C 1s peaks indicating the presence of zinc (Zn), oxygen (O), nitrogen (N) and carbon (C). The O 1s spectra of ZnO@ZIF-8, CNPs, and ZnO@ZIF-8/CNPs composite have oxygen species O_β_ and O_γ_ occurring at 531.8 and 533.1 eV respectively but ZnO@ZIF-8 and ZnO@ZIF-8/CNPs have an extra oxygen species positioned at O_α_ 529.3 eV. The oxygen at O_α_ is assigned for lattice oxygen species, O_β_ represents the surface adsorbed oxygen species and O_γ_ represents the adsorbed OH on the sensing materials.^36,57^Fig. 7e and f present the peaks at 1021.1 eV and 1044.5 eV assigned for Zn 2p_3/2_ and Zn 2p_1/2_ respectively, with separating binding energy of about 23.4 eV proving the existence of Zn^2+^ in ZnO@ZIF-8 and ZnO@ZIF-8/CNPs composite.^58^

### 3.2. Sensing studies

#### 3.2.1. Gas response, sensitivity, and selectivity

The electrical response was investigated during the interaction between all the prepared sensors, (sensor A, sensor B, sensor C, sensor D, sensor E, and sensor F) and the volatile organic compounds, *i.e.* methanol, ethanol, 2-propanol, acetone, chloroform, and ethyl acetate. All the sensing applications were done at room temperature and sensitivity investigations were done by varying the mass of ZnO@ZIF-8 nanorods within the CNPs/ZnO@ZIF-8 nanorods while the mass of CNPs was kept constant. The sensitivity of the sensors was considered to be equivalent to the gradient, *i.e.* “*S*” = Δ*R*/Δ*C*, where *S* represents sensitivity, Δ*R* is the relative response and Δ*C* is the concentration of the analyte. Sensor A, which was based on only ZnO@ZIF-8 nanorods, as a control, did not show any response during the exposure of any of the analyte vapours (for example for methanol see Fig. S6†), the non-responsive behaviour of the sensor at room temperature is due to the ZnO@ZIF-8 nanorods-based sensors can only respond at elevated temperature.^34^

**Fig. 8:**
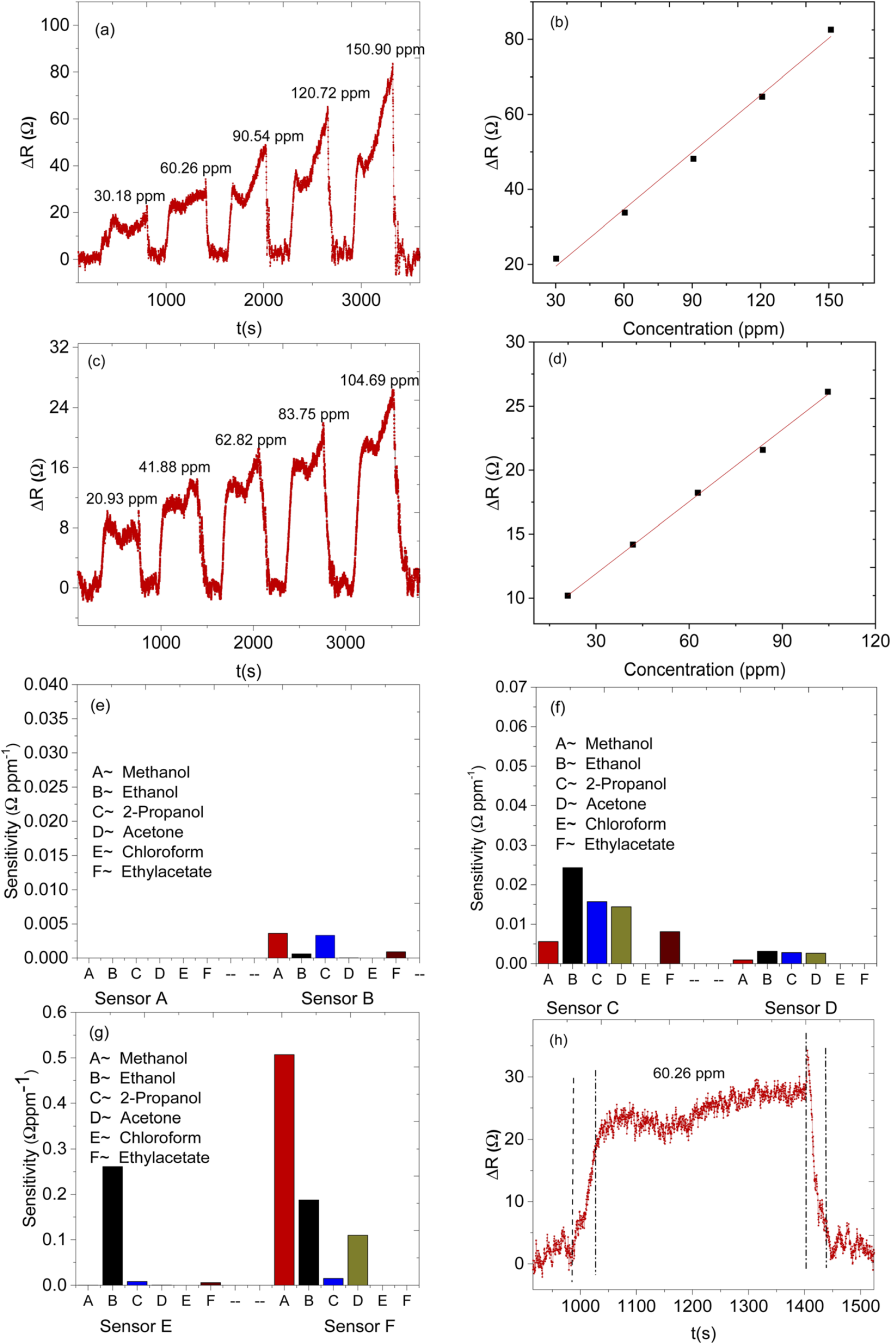
Dynamics response–recovery curve of sensor F (a) and (c) for methanol and ethanol vapour respectively and their corresponding calibration curve for methanol and ethanol (b) and (d) respectively. Response–recovery times of methanol on sensor F (d), the sensitivity of the sensors towards various organic vapour (e) for sensors A and B; (f) for sensors C and D; (g) for sensors E and F; (h) the response–recovery profile of sensor F towards methanol.

**Fig. 9:**
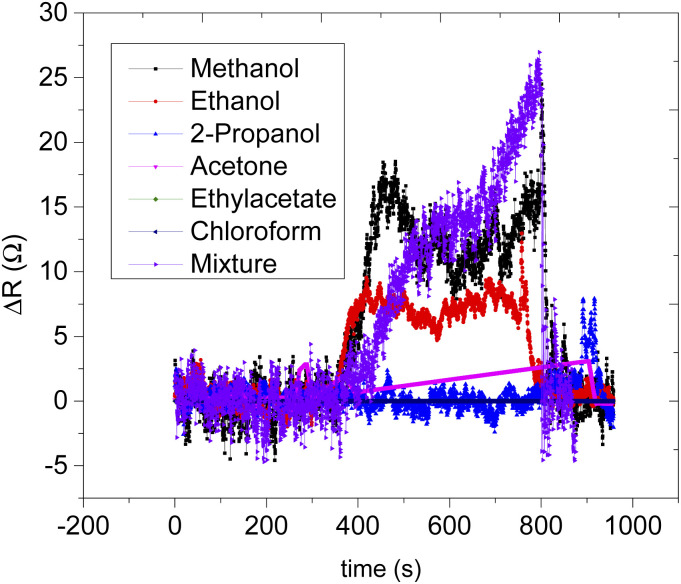
Static response–recovery curve of sensor F.

Sensor B (based on only CNPs) on the other hand responded to all the VOCs, however, there is a lack of selectivity to the specific analyte vapour as shown in (Fig. 8a) and (Fig. S1†). The remaining sensors, sensors C, D, E, and F, were all based on the composite mixture of CNPs and ZnO@ZIF-8 nanorods at different mass ratios. All the CNPs/ZnO@ZIF-8 composite-based sensors showed a high signal-to-noise ratio and showed a better performance than sensor A (see additional information). The increase in the performance of the sensors is due to the synergic effect of ZnO@ZIF-8 nanorods with the CNPs which enhanced the response of the analyte vapours. For all the sensors that showed good performances, the resistance of the sensors increased as the analyte vapour concentration increased and in most cases, a linear relationship was obtained between the sensor response and exposed analyte concentration (see Fig. 8b and c for example). A similar response was also recorded for sensor B (see Fig. S2†). Although the performance of the sensors has improved by mixing the two sensing materials, in terms of sensitivity, except for sensors E and F, all have lacked sensitivity towards specific analyte vapour (see Fig. 9g). According to the results, sensor F is highly sensitive toward methanol over the other tested analytes, the sensor's sensitivity toward methanol vapour was approximately 2.6 times more than to the ethanol vapour and it is 125 times more sensitive than sensor B. Sensor E is more sensitive toward ethanol than any other exposed analyte vapour, comparing sensor E with sensor F, sensor E is 30% more sensitive than sensor F. In terms of the response and recovery time toward methanol, sensor F is the best performing sensor in the response and recovery time as compared to the remaining sensors with a response time of 49 s and recovered back to its baseline in 47 s (see Table 2). The response and recovery time for the sensors that were exposed to ethanol vapour, generally all the responded sensors showed quick response and recovery time and sensor D was the fastest to respond and recover. Sensor E is the second-fastest to respond and recover, it took 37 s to respond and 39 s to recover to the baseline (see Table 3). Sensor F, however, was quick to respond and recover in the case of ethanol than methanol, 36 s to respond and 59 s to recover.

**Table 2:** Response and recovery time on the sensors by the methanol vapour^a^

Sensor	Mass ratios, CNPs : ZnO@ZIF-8 NRs	Response time (s)	Recovery time (s)
Sensor A	0 : 10	—	—
Sensor B	15 : 0	11	66
Sensor C	15 : 15	219	61
Sensor D	15 : 25	80	78
Sensor E	15 : 35	37	119
Sensor F	15 : 60	49	47

a No response is denoted by (—).

**Table 3:** Response and recovery time on the sensors by the ethanol vapour^a^

Sensor	Mass ratios, CNPs : ZnO@ZIF-8 NRs	Response time (s)	Recovery time (s)
Sensor A	0 : 10	—	—
Sensor B	15 : 0	63	98
Sensor C	15 : 15	38	45
Sensor D	15 : 25	31	34
Sensor E	15 : 35	37	39
Sensor F	15 : 60	36	59

a No response is denoted by (—).

Selectivity in the detection of methanol in the presence of a mixture (ethanol, 2-propanol, acetone, ethyl acetate, and chloroform) of vapours were studied (see Fig. 9). The sensor's response to the mixture analyte vapour was compared with the sensor's response to the individual analyte vapour (see Fig. 9). It can be seen that the methanol vapour had a maximum response of 24.7 Ω ppm^−1^ and followed by ethanol vapour which was 12 Ω ppm^−1^ then followed by acetone vapour was 5 Ω ppm^−1^ and 2-propanol vapour which was 3 Ω ppm^−1^. There were no responses recorded for ethyl acetate and chloroform vapours. Interestingly the sensor's response to the mixture of all the analytes vapour was measured as 27.6 Ω ppm^−1^ which was slightly over the response magnitude of the methanol, thus the response of the sensor towards the mixed analyte vapour is believed to be largely due to the presence of methanol in the mixture. The presence of other analyte mixture vapour did not affect much the sensor response towards methanol vapour. In terms of the response and recovery time, the presence of other analyte mixture, however, had influenced the response time of the sensor. Sensor F took twice the time to respond in the presence of mixture analyte vapours and the recovery time was as quick as the methanol analyte (see Table 4). A delay in response time might be due to the presence of other than methanol analytes vapour that competes for the active site of the sensing materials.

**Table 4:** Response and recovery times of the vapour analytes on sensor F^a^

Sensor F	Response time (s)	Recovery time (s)
Mixture	141	28
Methanol	49	47
Ethanol	70	30
2-Propanol	60	43
Acetone	81	64
Chloroform	—	—
Ethyl acetate	—	—

a No response is donated by (—).

#### 3.2.2. Sensor humidity effect and reproducibility

**Fig. 10:**
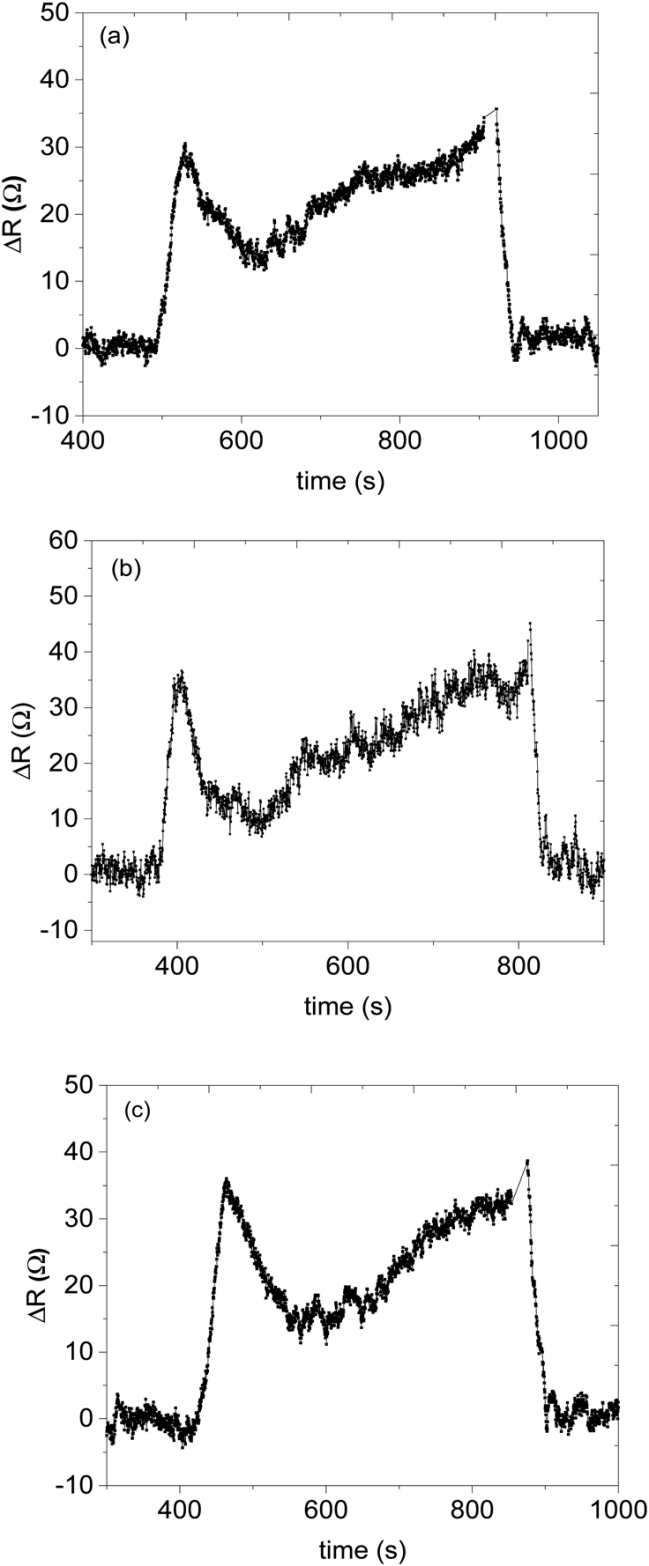
Methanol vapour response at RH (a) 33% (b) 53% (c) 61%.

**Fig. 11:**
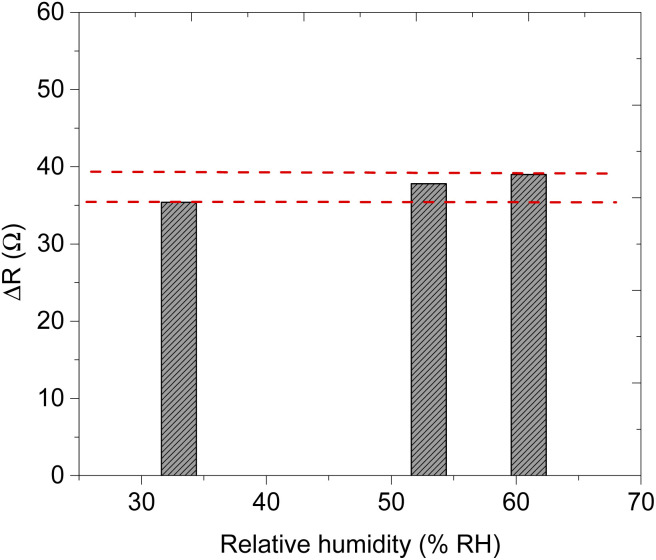
Methanol response–relative humidity bar graph.

**Fig. 12:**
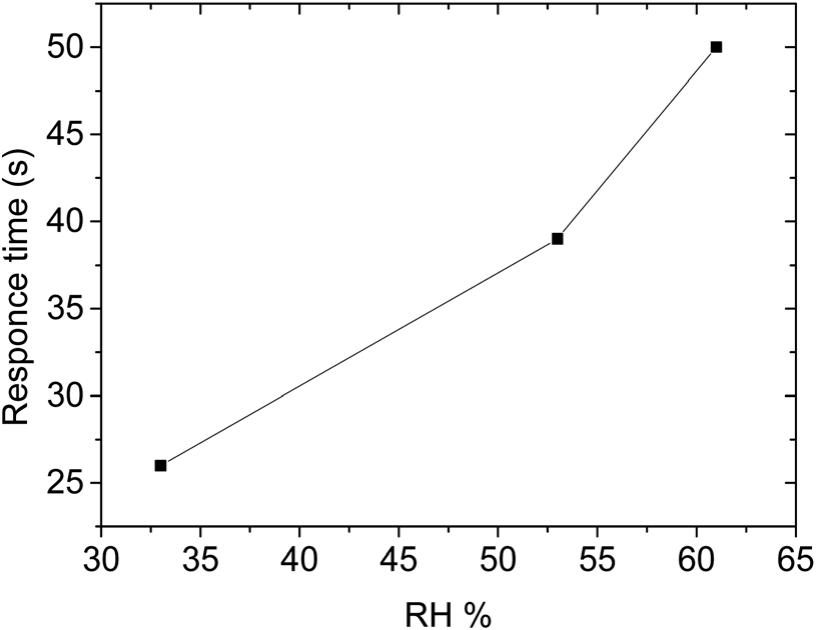
The response time *versus* relative humidity.

The presence of humidity during the detection of volatile organic compounds can affect the sensor's performance.^59^ Under humid conditions, the water molecules usually bind to the sensing materials and cause drifting of the sensor responses; and by occupying the active area of the sensing materials it blocks the analyte molecules not to interact with the sensing materials which leads to a decrease in the activities of chemisorption between the target gas with the metal oxide.^60^ It is also expected that decrease the sensitivity of the sensors and the baseline resistance of the gas sensor.^60^ Therefore it is important to study the effect of humidity on the performance of the fabricated sensor. The effect of humidity during the detection of methanol using sensor F was studied at various relative humidity (RH) of 33%, 53%, and 61% with about 60 ppm of methanol vapour in the system (see Fig. 10). The maximum response of methanol at these respective RH was 35.4 Ω, 37.8 Ω, and 39.2 Ω. Interestingly, as the RH increased from 33% to 61% which is about twice the initial value, the response of the sensor changed only by 5%. The response–recovery curve shape has remained the same at different humidity, rises during interaction and falls back to the baseline when the analyte vapour is removed. This can be evidence that the fabricated sensor is not affected much by the presence of water molecules in the system (see Fig. 11). Interestingly, the humidity effect was profoundly observed in the response time as the humidity concentration increased from 33% to 61% we observed a delay in response time approximately double from 26 s to 50 s (see Fig. 12). However, the recovery time of the sensor F was almost the same over the range (see Fig. 10 and Table 5).

**Table 5:** Response and recovery times of methanol vapour on sensor F at different humidity conditions

Relative humidity (RH) (%)	Response time (s)	Recovery time (s)
33	26	19
53	39	19
61	50	22

#### 3.2.3. The sensing mechanism

The ZIF-8 layer works as a separator or filter to allow methanol vapour to pass through to reach ZnO nanorods. In the case of metal oxide (ZnO nanorods), semiconductor metal oxides (SMOs) including TiO_2_,^16^ WO_3_,^19^ ZnO, and SnO_2_ (ref. 61) use an adsorption–desorption sensing mechanism. At high temperature, it is expected that free electron flows easily through the grain boundaries of SMO films. When the sensor is exposed to an oxygen atmosphere, the oxygen molecules are adsorbed on the SMO surface. The adsorbed oxygen molecules on the surface of the SMO surface trap electrons from the bulk of the materials and form a layer of charged reactive oxygen species (O_2_^−^, O_2_^2−^, O^2−^).^62^ Those reactive oxygen species repel other electrons not to interact with the sensing materials, resulting in a region where depleted electrons and increased potential barrier at the grain boundaries. This restricts the flow of electrons through the sensing materials and increases the resistance of the sensor.^63^

During the sensing process for the n-type semiconductors, the analyte molecules adsorb on the surface of the sensing materials. In the case of reducing analyte molecules (for example methanol), the molecules on the surface reduce the potential barrier by injecting the trapped electrons back into the conduction band and allowing the electron to flow easily as a consequence, as a result, the sensors' resistance reduces. It is the opposite for oxidising analyte molecules^64^ as the analyte vapour reacts with reactive oxygen species it produces carbon dioxide and water molecules as by-products.^65,66^ The more availability of adsorbed atmospheric oxygen molecules facilitates the surface reaction with the analyte molecules, improving the sensing performances. Usually, during VOCs sensing at room temperature, the O_2_^−^ reactive species are responsible for the total decomposition of VOCs into CO_2_ and H_2_O molecules. However, at high temperatures, the O_2_^2−^ and O^2−^ reactive species become very active and these reactive species tap more electrons as compared to O.^24,35,36^

For p-type SMOs gas sensors, oxygen molecules from the atmosphere are adsorbed on the surface of the SMOs and, under ambient conditions, oxygen (O_2_) molecules react with the electrons and become O_2_^−^ on the surface of SMOs and holes are accumulated and form hole accumulation layer (HAL) with low resistance.^67^ When the sensors are exposed to reducing gases, electrons are injected back into SMOs and decrease the concentration of holes in HAL and as a consequence, the resistance of the sensor increase. Interestingly, sensor B, the CNPs-based sensor, responded well during the exposure of the sensor to methanol and ethanol vapours (see Fig. S1†). As it is exposed to the reducing analyte molecules, the resistance of the sensor increases and when the analyte molecules are removed from the system and the sensors are exposed to atmospheric air the resistance returns back to the lowest value which means high conductivity. This behaviour is consistent with the p-type semiconductor electrical conductivity. From our previous studies, the CNPs are involved in the total decomposition of VOCs into CO_2_ and H_2_O,^68,69^ this is due to the presence of reactive O_γ_ and O_β_ reactive species on the surface of CNPs (see Fig. 7b)^36^ which are active at room temperature. Reports on the carbon nanomaterials-based sensors indicated that when the sensors are exposed to an electron acceptor such as NO_2_ molecules, causes a decrease in the resistance of the sensor^70^ and on the contrary when the sensors are exposed to electron donors such as ethanol, the resistance of the sensor increases.

Generally, a report has shown that the ZIFs-based sensors' behaviour display p-type semiconductor electrical conductivity,^71^ in our case, we form n–p types of heterojunctions by synthesising the ZnO@ZIF-8, and nevertheless, the sensor didn't respond when exposed to any of the analyte vapours at room temperature and there was a high noise-to-signal ratio during the measurement. However, the introduction of CNPs into ZnO@ZIF-8 nanorods allowed the formation of p–n–p-type heterojunctions, but the behaviour of the sensor was dominated by the p-type. As the sensor is exposed to the methanol vapour, the sensor's resistance increased and when the analyte vapour is removed and the sensor is exposed to the atmospheric air, the sensor conductivity increase. The incorporation of the CNPs allows the sensor to perform better at room temperature by improving the electrical conductivity resulting in decreasing the resistance of the sensor^72^ as well as the synergistic effect between the two sensing materials. The proposed reaction equation is as follows:

1 O_2_(gas) + e^−^ ↔ O_2_^−^(ads)

2 O_2_^−^(ads) + e^−^ ↔ O_2_^2−^(ads) ↔ 2O^−^(ads)

3 CH_3_OH + 3O^−^(ads) → CO_2_ + 2H_2_O + 3/2e^−^

During the interaction of the analyte vapour with the sensor, the electrical resistance of the sensor increases as a result of the interaction between the methanol vapour and the adsorbed reactive oxygen species resulting in the total decomposition of the VOCs.

#### 3.2.4. Limit of detection

The limit of detection (LOD) is the lowest possible quantity of concentration that can be detected by the sensor. The relationship between electrical response and methanol vapour concentration between 30 to 150 ppm was approximately linear, the correlation coefficient (*R*^*2*^) and slope of the linear fit were 0.99 and 0.51 Ω ppm^−1^ respectively. LOD can be calculated using the formula LOD = 3 × RMS/slope,^73^ wherein RMS was the standard deviation and the LOD was found to be 0.001, LOD was approximated to be 60 ppb, which highlights that the sensor can detect very low concentration.

## 4. Conclusions

In summary, the successful synthesis of CNPs and ZnO@ZIF-8 nanorods and CNPs/ZnO@ZIF-8 nanorods composites were confirmed by SEM, TEM, XRD, FTIR, XPS and Raman spectroscopy. All synthesized materials were used to fabricate the sensor, and among all prepared sensors, sensor F was found to be highly sensitive and selective towards the detection of methanol vapour over ethanol, acetone, 2-propanol, ethyl acetate, and chloroform. The introduction of carbon nanoparticles prepared from pyrolysis reaction into ZnO@ZIF-8 nanorods to form CNPs/ZnO@ZIF-8 nanorods drop the high working temperature to room temperature. The formation of composites able to form p–n–p type of heterojunction but the sensor's behaviour was p-type semiconductor electrical conductivity. According to our obtained results, sensor F shows a fast response–recovery time, and the LOD was found to be 60 ppb. In addition, sensor F shows almost the same results at various humidity conditions with a methanol vapour response error of about ±5%. Carbon material/semiconductor metal oxide@ZIF composites are promising novel materials to enhance gas sensing performances, no humidity impact and to reduce the high known working temperature of the gas sensors to room temperature.

## Author contributions

L. Malepe performed all the experiments, and investigations and wrote the manuscript. M. A. Mamo conceived the idea, and guidance and reviews the manuscript. P. Ndungu and D. T. Ndinteh are responsible for providing resources, editing, and reviewing the manuscript.

## Conflicts of interest

There are no conflicts to declare. The manuscript was written through the contributions of all authors.

## Supplementary Material

RA-012-D2RA04665B-s001
