# Supplementary material for: Selective detection of methanol vapour from a multicomponent gas mixture using a CNPs/ZnO@ZIF-8 based room temperature solid-state sensor
Source: RSC Adv. 2022 Sep 23;12(42):27094–108. doi: 10.1039/d2ra04665b (PMC9501866; doi:10.1039/d2ra04665b)
Supplement: RA-012-D2RA04665B-s001 [file RA-012-D2RA04665B-s001.pdf]

Electronic Supplementary Material (ESI) for RSC Advances.
This journal is © The Royal Society of Chemistry 2022

Selective detection of methanol vapour from a multicomponent gas mixture using a CNPs/ZnO@ZIF-8 based room temperature solid-state sensor

Lesego Malepe, [a] Derek Tantoh Ndinteh, [a] Patrick Ndungu *[+b] and Messai Adenew Mamo *[a]

[a] *Department of Chemical Science, University of Johannesburg, PO Box 17011, Doornfontein, 2028 Johannesburg, South Africa*

[b] *Department of Chemistry, University of Pretoria, Private Bag X20, Hatfield, 0028, Pretoria, South Africa*

Supporting information

(a) Response of sensor B (Sensor-based on only CNPs)

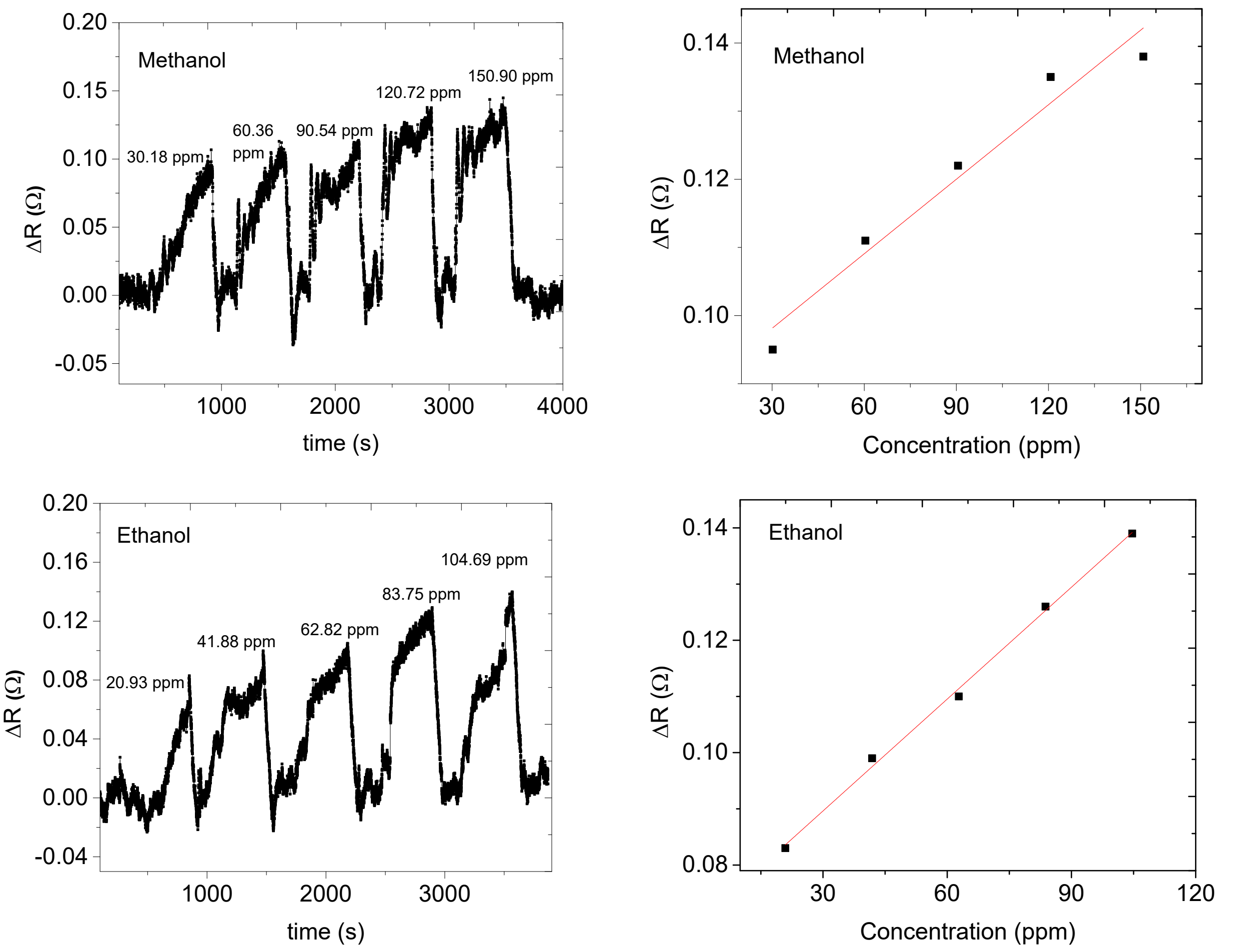

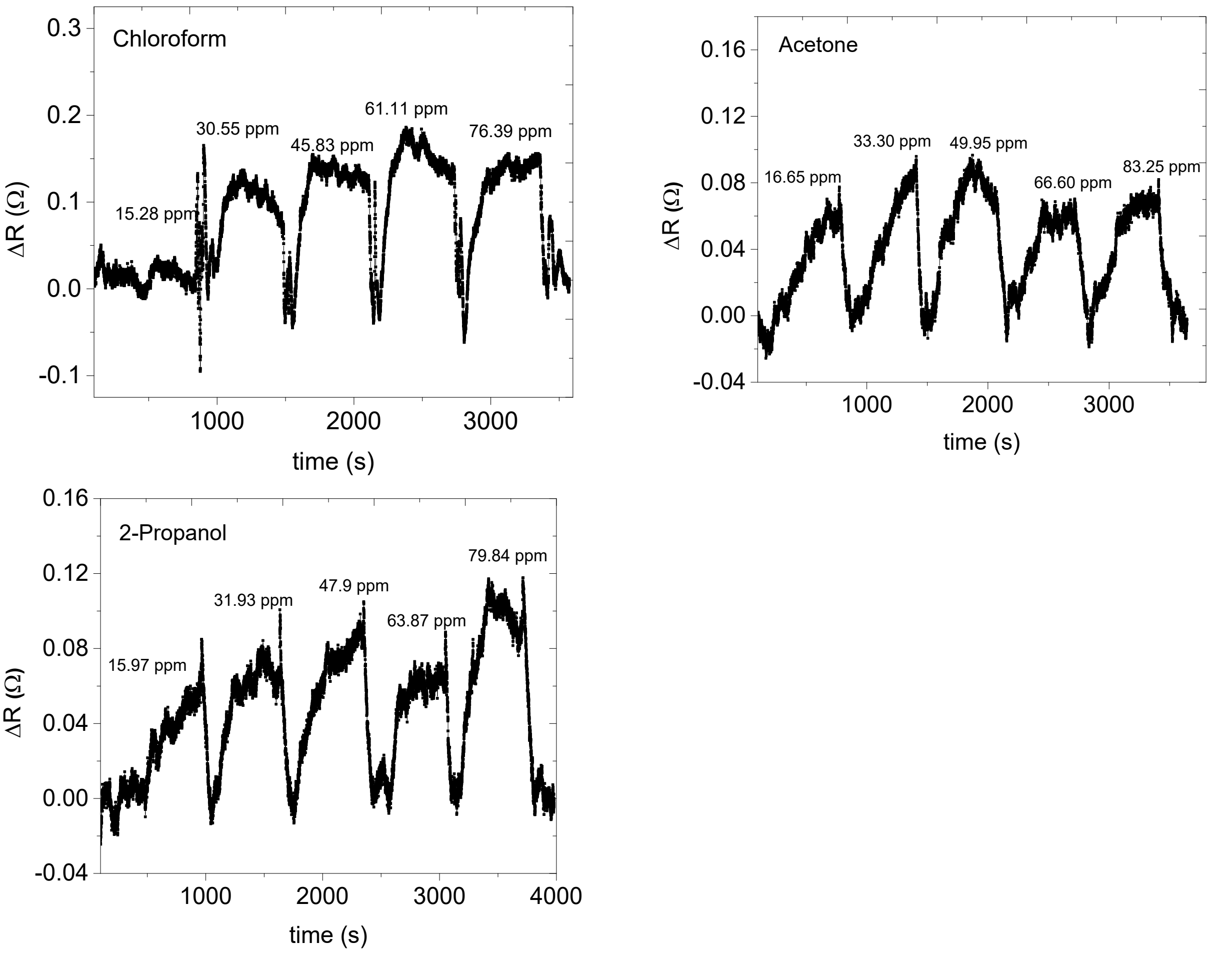

Fig. S1: Dynamic response and recovery curve of the sensor based on CNP only (Sensor B).

(b) Sensor C CNPs/ZnO@ZIF-8 (15: 15)mg

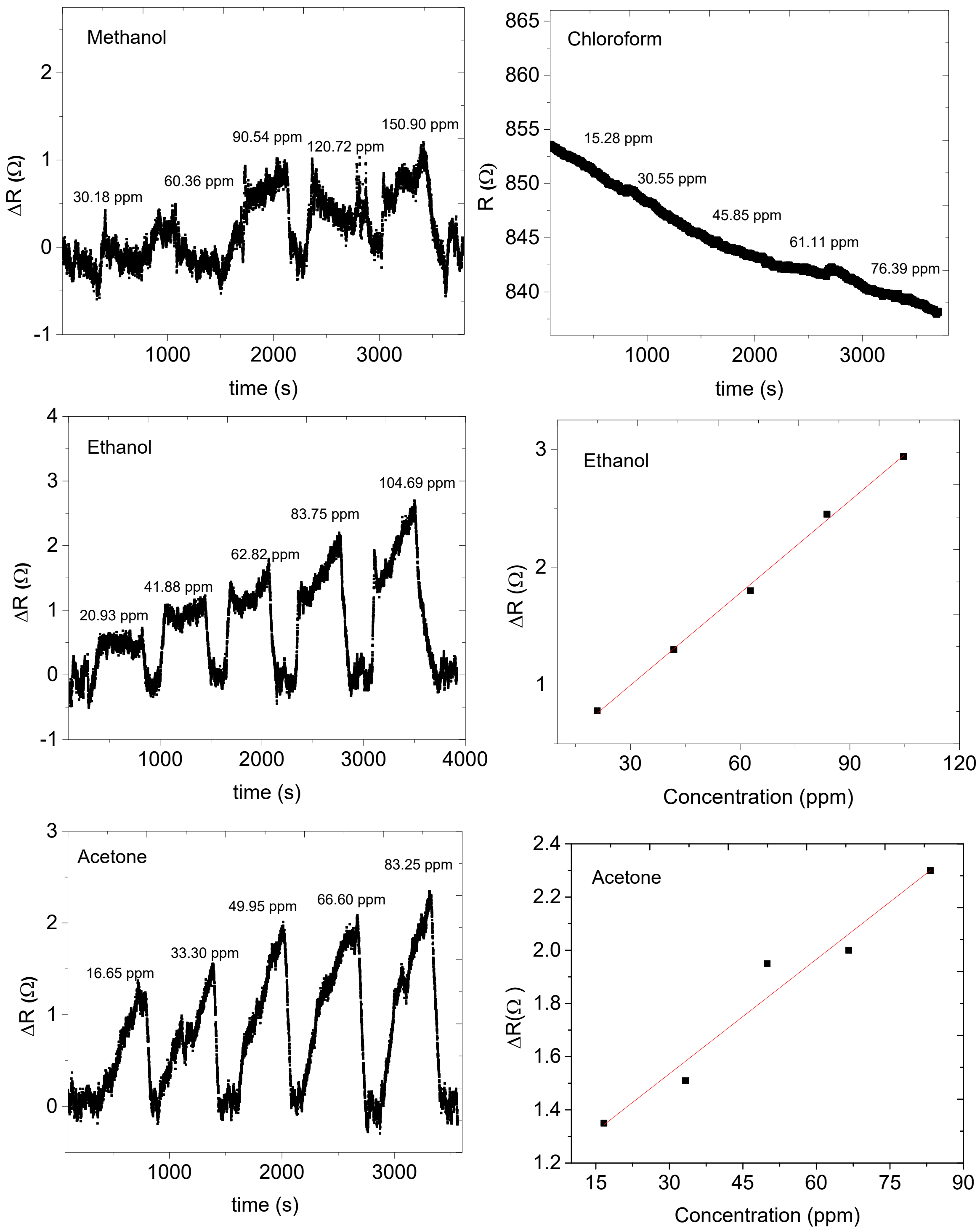

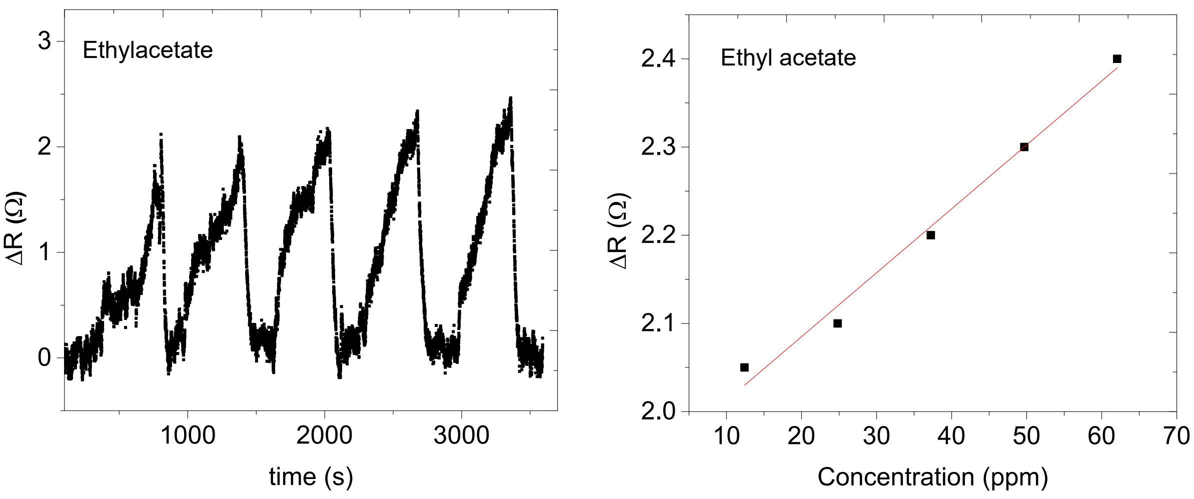

Fig. S2: Dynamic response and recovery curve of the sensor based on CNPs/ZnO@ZIF-8 (15:15) mg sensor (sensor C)

(c) Sensor D CNPs/ZnO@ZIF-8 (15:25) mg

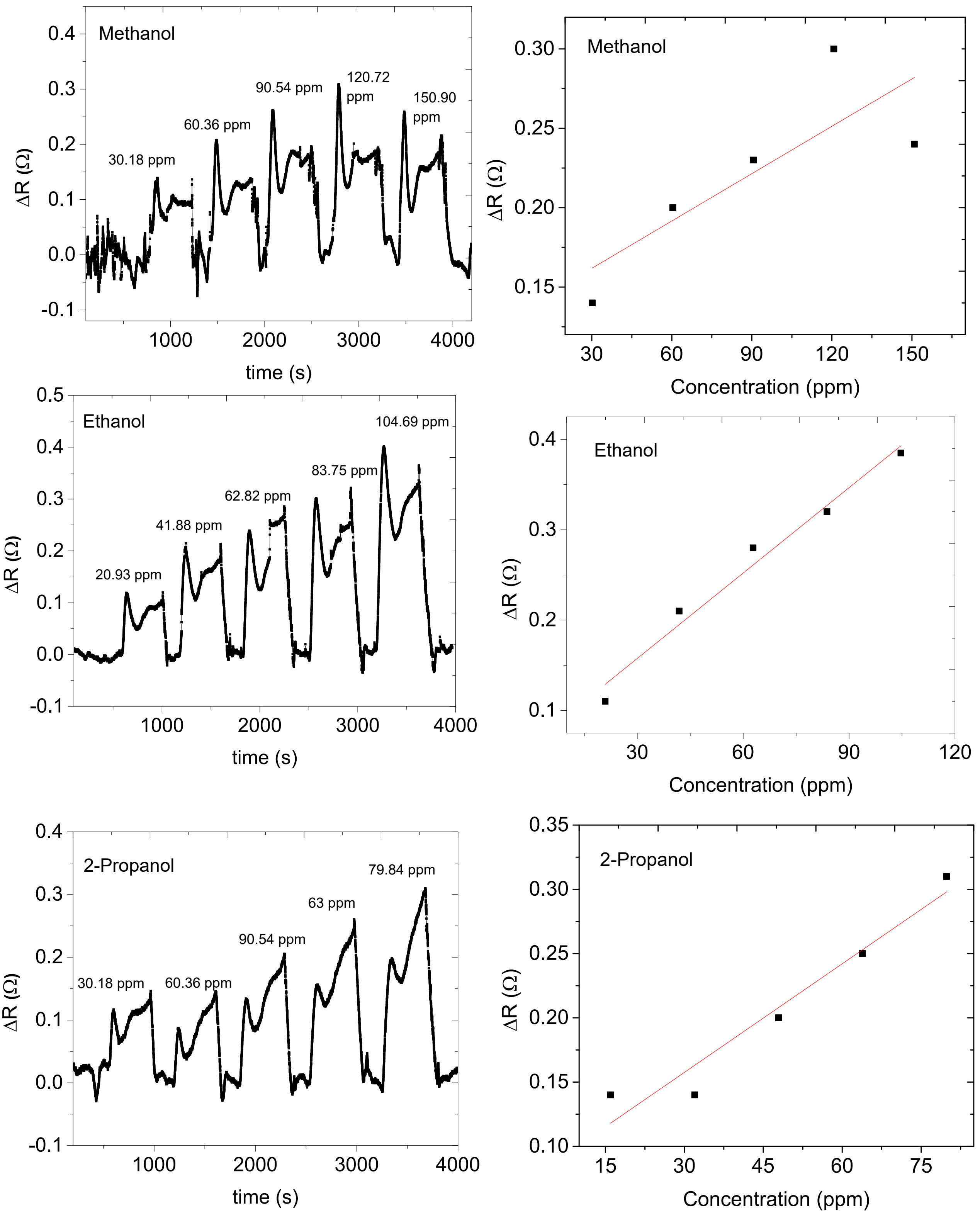

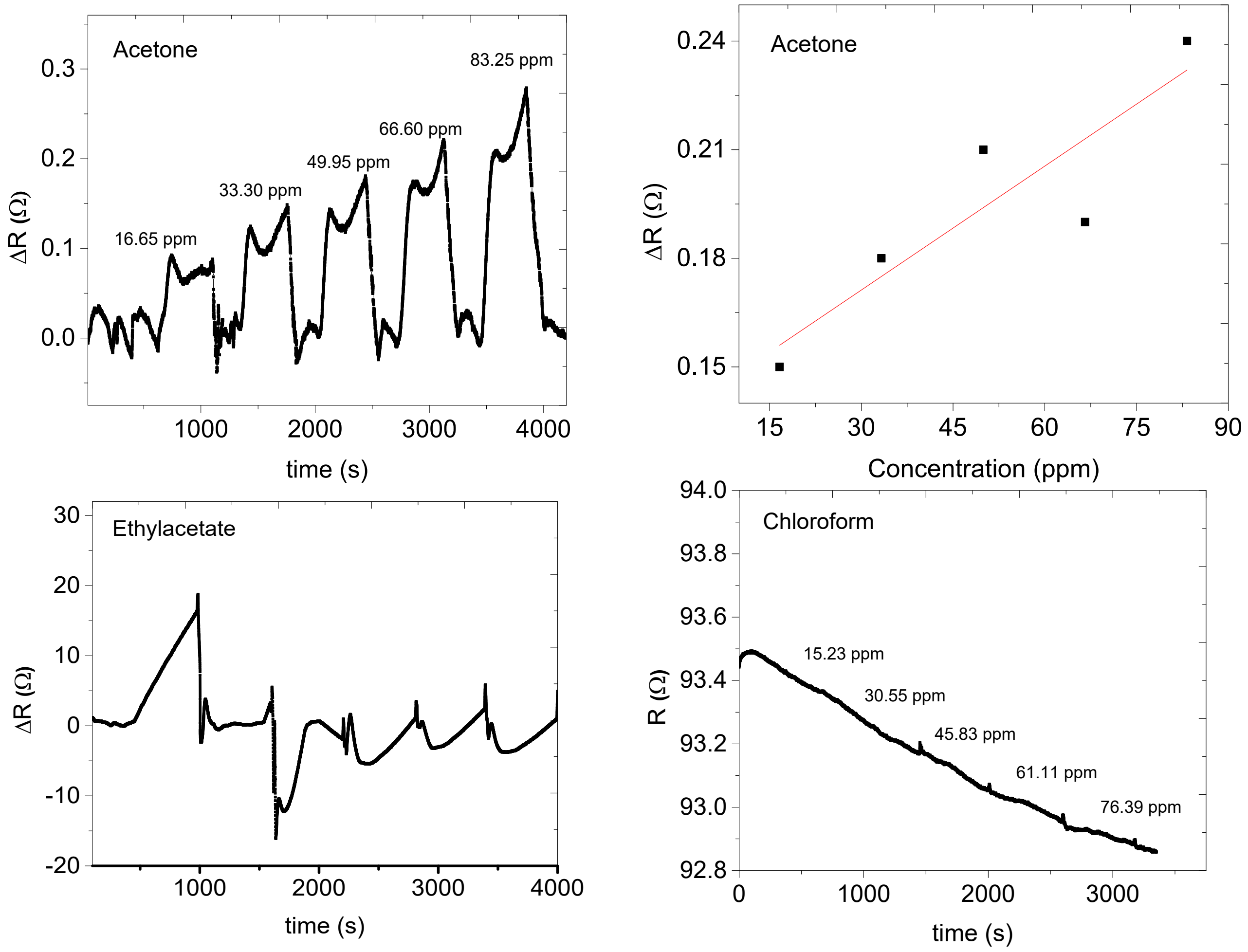

Fig. S3: Dynamic response and recovery curve of the sensor based on CNPs/ZnO@ZIF-8 (15:25) mg sensor (sensor D).

(d) Sensor E CNPs/ZnO@ZIF-8 (35:15) mg

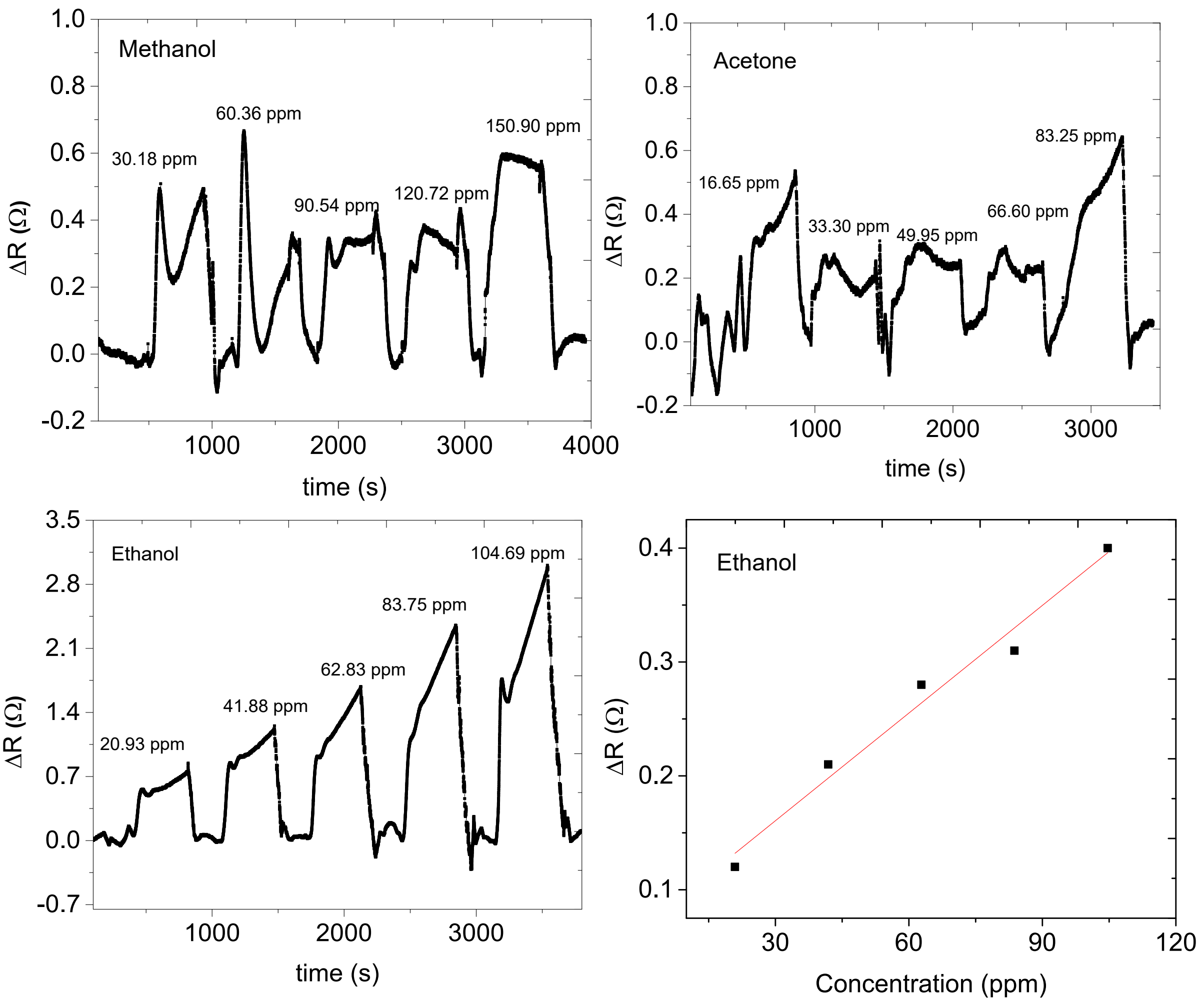

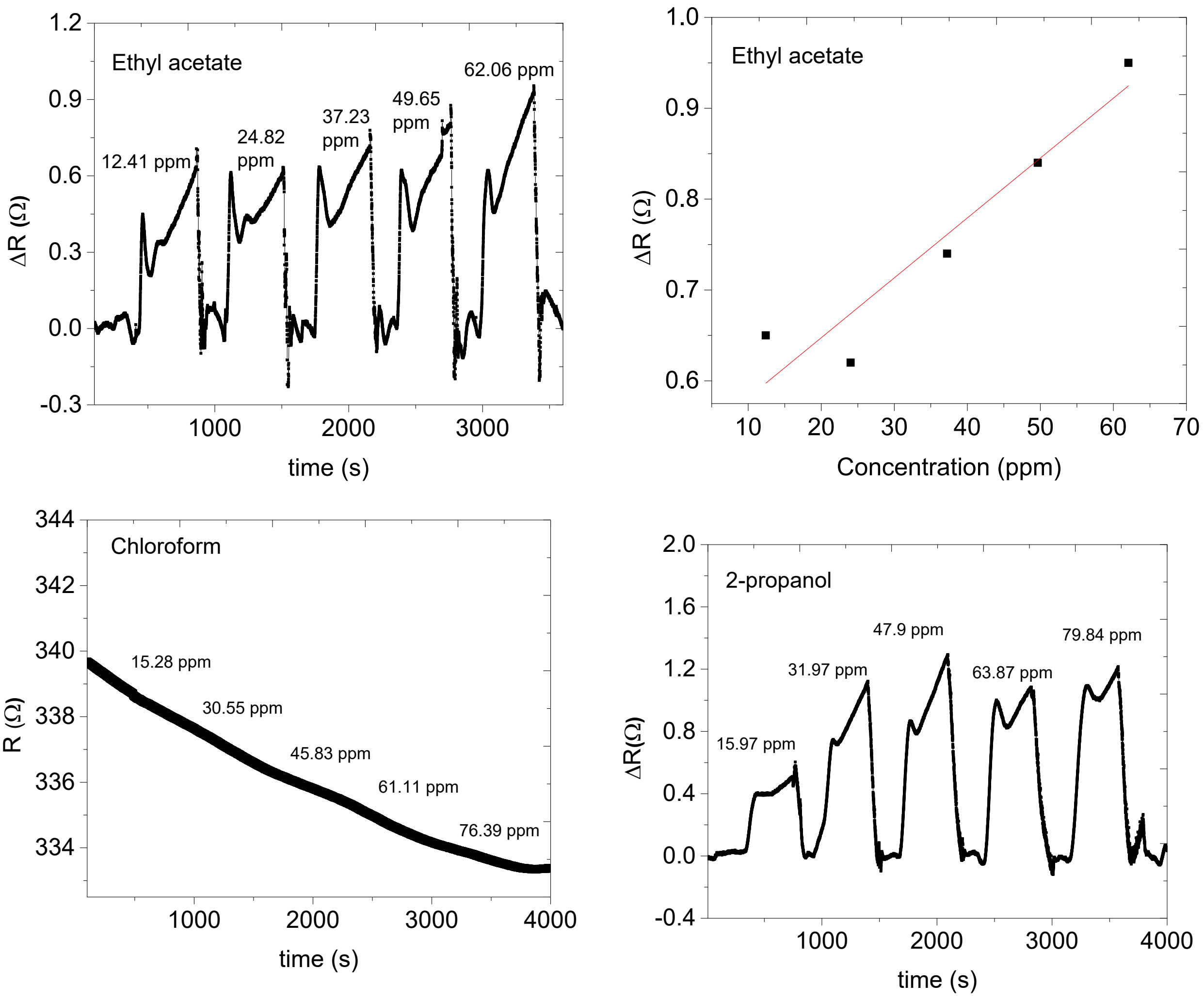

Fig. S4: Dynamic response and recovery curve of the sensor based on CNPs/ZnO@ZIF-8 (15:35) mg sensor (sensor E).

(e) Sensor F CNPs/ZnO@ZIF-8 (15:60)mg

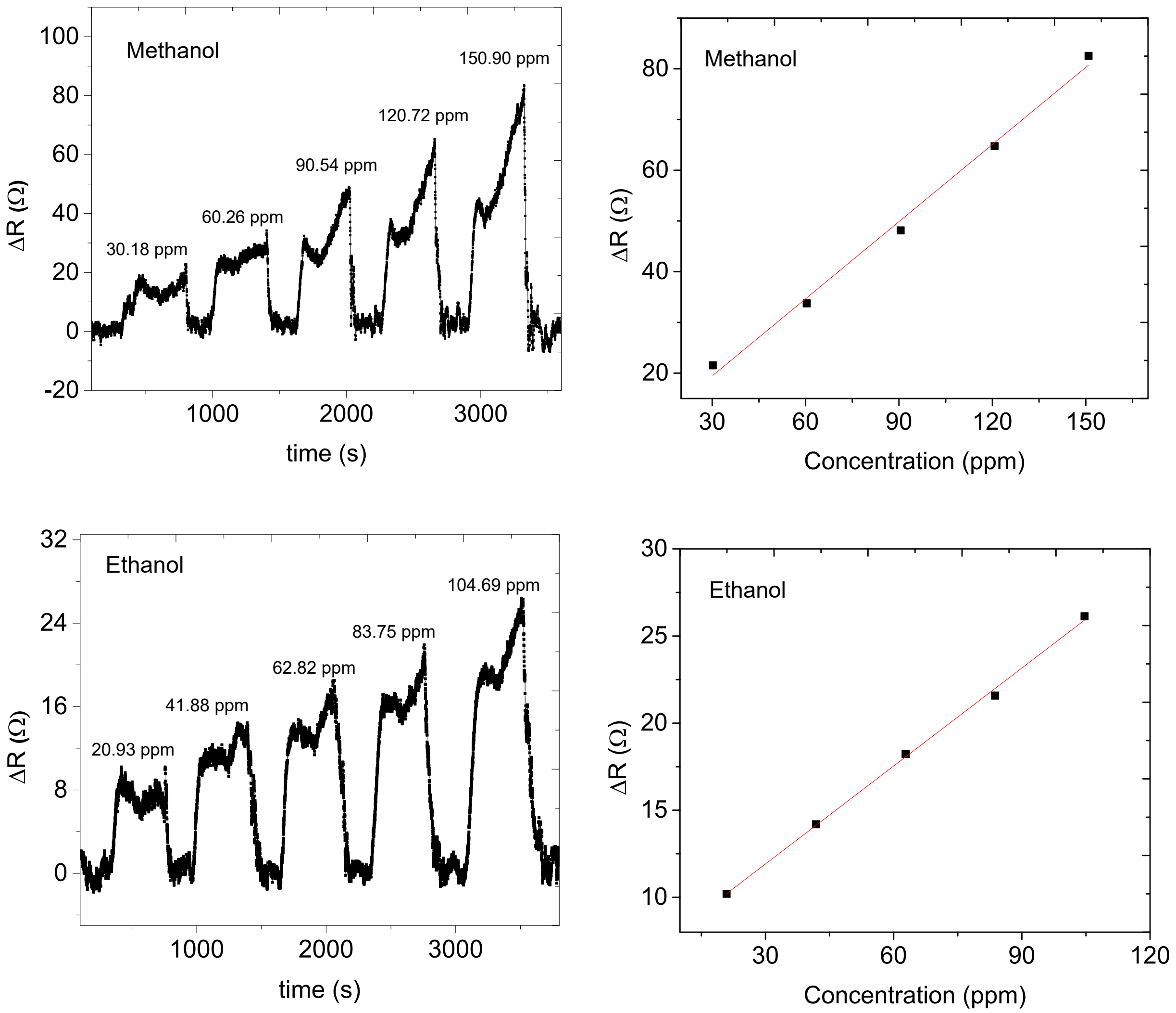

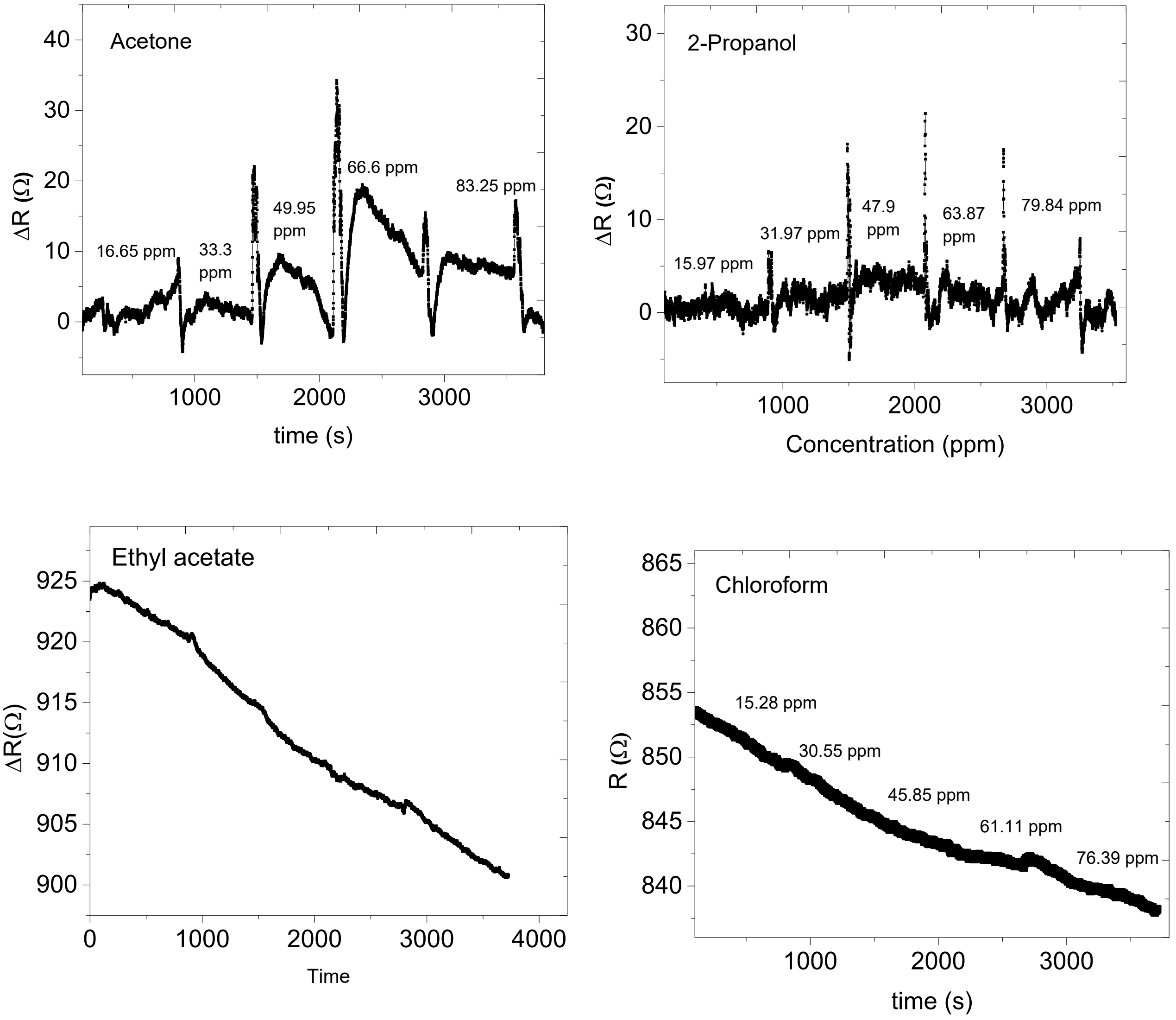

Fig. S5: Dynamic response and recovery curve of the sensor based on CNPs/ZnO@ZIF-8 (15:60) mg sensor (sensor F)

(f) the sensor based on ZnO@ZIF-8 towards methanol

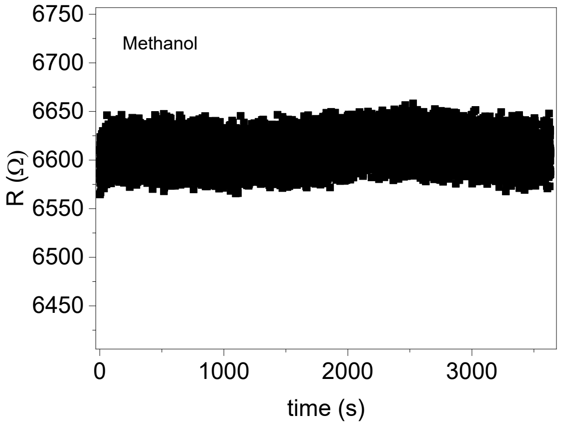

Fig. S6: Dynamic response and recovery curve of the sensor based on ZnO@ZIF-8
